# Modeling the impact of L2 grit on EFL learners’ core of self-assessment and foreign language anxiety

**DOI:** 10.1186/s40468-022-00200-6

**Published:** 2022-11-04

**Authors:** Tahereh Heydarnejad, Sayed M. Ismail, Goodarz Shakibaei, Abdulbaset Saeedian

**Affiliations:** 1 Department of English Language, Faculty of Literature and Humanities, University of Gonabad, Gonabad, Iran; 2 College of Humanities and Sciences, Prince Sattam Bin Abdulaziz University, Al-Kharj, Saudi Arabia; 3 Department of English, Faculty of Humanities, Ahvaz Branch, Islamic Azad University, Ahvaz, Iran; 4 Department of Foreign Languages, TUMS International College, Tehran University of Medical Sciences, Tehran, Iran

**Keywords:** L2 grit, Core of self-assessment, Foreign language anxiety, EFL learners, SEM, Effective language instruction and assessment

## Abstract

Learners’ personality traits and self-assessment have an essential role in their academic achievement and the well-being of society. Although L2 grit and the core of self-assessment (CSA) have attracted considerable attention in educational research, few studies have focused on the impact of L2 grit on boosting CSA and managing foreign language anxiety (FLA). Drawing upon this existing research gap, the present study set forth to test a structural model of English as a Foreign Language (EFL) university learners’ L2 grit, CSA, and FLA. The language-domain-specific grit scale (LDSGS), the core of self-assessments questionnaire (CSAQ), and the Foreign Language Classroom Anxiety Scale (FLCAS) were administered to 418 Iranian EFL university learners. The findings of structural equation modeling (SEM) reflected the contributions of L2 grit and CSA to overcoming language learners’ experienced anxiety. Furthermore, the significant influence of EFL learners’ CSA on FLA was concluded. The implications of the findings are to raise learners’ awareness of their personality traits and self-assessment that can foster effective language instruction and assessment.

## Introduction

Learners’ L2 grit is an amalgamation of perseverance of attempt and passion for long-term goals. Previous studies in the realm of grit reflected that this construct is significantly related to other teacher and student-related constructs, leading to the success of education (Shafiee Rad & Jafarpour, 2022; Steinmayr et al., 2018; Sudina et al., 2021; Sudina & Plonsky, 2021; Vadivel & Beena, 2019; Zheng et al., 2022). More specifically, the success of students in language learning is highly dependent on their effort and their passion for long-term goals; thus, L2 grit and its contribution to language assessment and academic achievement are of great importance. As Dale et al. (2018) and Lan (2022) stipulated, individuals with high levels of grit have a positive viewpoint about their professional lives. From another perspective, grit as a personality trait empowers individuals to direct their energies and distinguish the difference between high-priority and low-priority objectives (Azizi et al., 2022; Hejazi & Sadoughi, 2022; Shirvan & Alamer, 2022; Vadivel et al., 2021). Grit is considered as significant as talent and can guarantee the learners’ success and their productivity beyond their natural or inherent capabilities (Duckworth et al., 2007; Jamali Kivi et al., 2021; Kolganov et al., 2022; Liu et al., 2021; Teimouri et al., 2020).

CSA is an integrated personality structure that is manifested in the students’ evaluation of their abilities. This notion mirrors the learners’ fundamental beliefs about themselves and their learning procedures (GuoJie, 2021; Tavousi & Pour Sales, 2018; Umeanowai & Lei, 2022). Students with high levels of CSA are more engaged in learning processes because a positive CSA triggers positive attitudes toward life experiences and boosts life satisfaction (Miller Smedema et al., 2015; Namaziandost et al., 2022; Özerl et al., 2016; Zhuoyuan, 2021). Positive CSA also leads to learners’ positive perspectives in challenging situations. In other words, high levels of self-assessment immunize learners against different challenges that they may experience in their educational lives (Kammeyer-Mueller et al., 2009). As the findings of the previous studies present, positive CSA in learners help them manage their emotions and have better social relationships with other people, especially their teachers and peers (Abdollahi et al., 2022; Sifatu et al., 2020; Wongdaeng, 2022).

Emotion is an inevitable part of students’ educational lives which affects their learning and assessment. Emotion and cognition are interwoven (Li et al., 2022). Among all the pleasant and unpleasant emotions that students may experience, anxiety is the most experienced negative emotion (Khajavy & Aghaee, 2022; Khajavy et al., 2020; Shafiee Rad & Jafarpour, 2022). As Oteir and Al-Otaibi (2019) stipulated, people may feel anxious when they are powerless. In line with this experienced negative emotion (i.e., anxiety), the concept of FLA was introduced, which concentrates on the anxiety that may foreign language learners experience (Burić et al., 2016; Richards, 2020). FLA is situation-specific anxiety that affects language learners’ self-perceptions, attitudes, and behavior, as well as their feelings in language classes (Bielak, 2022; Horwitz et al., 1986; Rezai et al., 2022a; Rezai et al., 2022b).

**Fig. 1:**
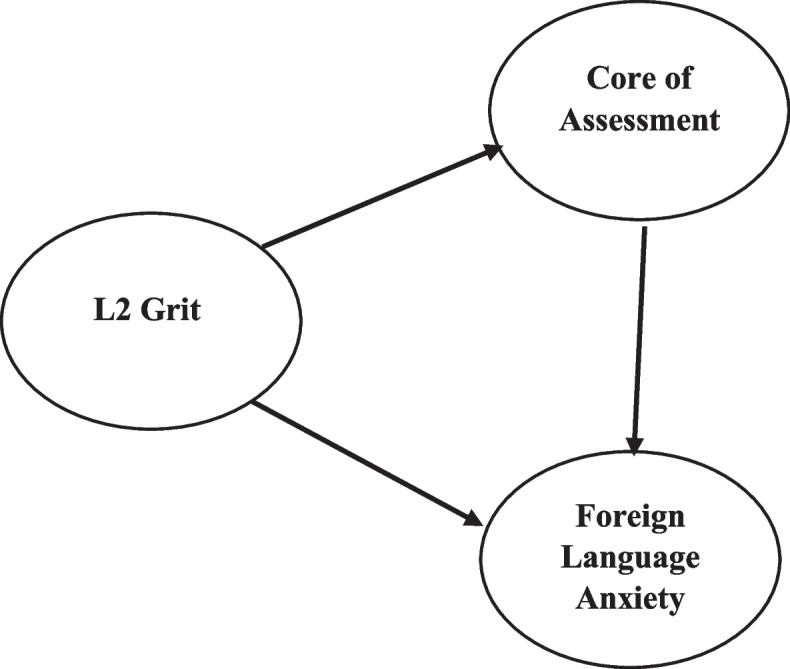
Theoretical structural equation model

Although learners’ L2 grit, CSA, and AFL play significant roles in language learners’ academic achievement, to the best of the researchers’ knowledge, no study has ever tried to investigate the interplay between these learner-attributed-construct. To this end, the researchers of the present study proposed a model to depict the impact of L2 grit on EFL learners’ CSA and FLA (See Fig. 1), and in this regard, the suggested hypotheses were tested. The following section intends to review the related literature and theories on L2 grit, CSA, and FLA.

### Literature review

#### L2 grit

The metaphor of grit refers to “working strenuously toward challenges, maintaining effort and interest over the years despite failure, adversity, and plateaus in progress” (Duckworth et al., 2007). In this regard, Duckworth et al. (2007) stipulated that besides talent, which is critical in the improvement of learners, grit also leads to their academic achievement. Learner L2 grit was theoretically proposed by Duckworth (2016) with emphasis on individuals’ enthusiasm and persistent attempts in their selected goals. Enthusiasm refers to individuals’ eagerness and desire to achieve a specific objective. Persistence as a critical part of grit inspires people to dedicate themselves to the processes of a particular objective (Duckworth, 2016). To clarify the nature of grit, Cormier et al. (2019), Teimouri et al. (2020), and Yang et al. (2022) claimed that grit is a domain-specific constitute. Considering this aspect of grit, Teimouri et al. (2020) proposed and validated a scale entitled, “the Language-domain-specific grit” to measure grit in language classes.

Grit in learners helps them to manage their stress and anxiety, leading to positive attitudes toward job affairs and their engagement (Zheng et al. (2022); Sudina et al., 2021). Due to its importance and support in the individual well-being, different educational organizations attempted to plan, design, and implement instructional materials and syllabi to apply learners’ grit as part of their educational programs (Cohen, 2015; Shechtman et al., 2013; Sutarto et al., 2022). Grittier students are more energetic, and they devote more time to their learning activities (Khajavy & Aghaee, 2022; Sudina & Plonsky, 2021). More precisely, students with high levels of grit can preserve their wish for long-term objectives even they face problems and challenges (Xu et al., 2022; Yang, 2021; Yang et al., 2022).

Leafing through the literature on learner grit mirrors recent attention to reciprocal relationships between students’ grit and its correlates in the educational domain. For instance, Wei et al. (2020) confirmed a positive association between L2 grit and English language achievement. Changlek and Palanukulwong (2015) conducted a study among Thai students to explore the motivational characteristics of gritty students. Based on their findings, grittier learners are more motivated and less anxious. In another study, Lee and Jang (2018) evidenced that grit, hope, growth mindset, and self-directed learning were positively related. Furthermore, they concluded that hope predicts the association between grit and the growth mindset. Taking a similar path, Wang et al. (2021) found that grit and the growth mindset are significantly related. Moreover, Cheng (2021) concluded that language-specific grit and future self-guides trigger language learners’ willingness to communicate. Additionally, Liu (2022) also confirmed the mediator role of EFL students’ academic motivation and growth mindsets in their grit. Shafiee Rad and Jafarpour (2022) documented that effective emotion regulation acts in favor of EFL learners’ L2 grit, ER, and resilience. Ghanbari and Abdolrezapour (2021) also found that EFL learners’ L2 grit and pleasant emotions were helpful in their academic success. Thus, it can be inferred that L2 grit and a balance in the emotional states of the learners influence the whole process of language development.

#### The Core of Self-assessment (CSA)

Assessment refers to the procedures involved in gathering, exchanging, and negotiating information from different relevant sources with the aim of providing a comprehensive understanding of what students know and need to learn (Bachman, 2015; Ebadi & Rahimi, 2019). Based on Bachman et al. (2010), “self-assessment refers to assessment or evaluation of oneself or one’s actions, attitudes, or performance. That is why each learner should be encouraged and trained to go through a process of self-assessment”. As Locke et al. (1996) defined, the core of self-assessment refers to the fundamental appraisal of one’s worthiness, effectiveness, and capability as a person. This notion is a broad, latent, higher-order trait that involves self-esteem, generalized self-efficacy, neuroticism, and locus of control (Judge et al., 1997). Self-esteem refers to the general values that individuals consider for themselves. Generalized self-efficacy is related to individuals’ evaluations of their performance in different situations (Locke et al., 1996). Neuroticism refers to the individuals’ attitudes toward a negativistic cognitive/explanatory style of thinking (Watson, 2000). Locus of control is related to individuals’ beliefs about the reasons for happenings (Judge et al., 1997).

Individuals with high levels of self-evaluation can cope with new situations and try to do their best in their responsibilities (Al-Mamoory & Abathar Witwit, 2021). High levels of self-assessment enable individuals to stop, think, and modify their emotional experiences (Putro et al., 2022). Learners who have developed a positive core of self-assessment manage their emotional experiences to improve their learning (Hu, 2022; Punpromthada et al., 2022; Rouhollahi et al., 2020; Snyder et al., 2012). In this regard, Eysenck (1990) argued that CSA can be assumed as a measure of emotional stability. More importantly, self-assessment supports learners’ well-being (Jahara et al., 2022). To implement self-assessment, learners should practice metacognitive skills (Wei, 2020), critical thinking (Zhang, 2022), reflective thinking (Davoudi & Heydarnejad, 2020), self-efficacy beliefs (Namaziandost & Çakmak, 2020), and academic emotion (Khajavy, 2021; Khajavy et al., 2020; Pekrun et al., 2002). In a recent attempt, Nemati et al. (2021) set forth a study to evaluate the role of self-assessment, peer assessment, and teacher assessment on writing strategy development among EFL learners. As the findings indicated learners who practice self-assessment show cognitive and metacognitive development during their research project. In line with this result, Jahara et al. (2022) explored the influence of coping styles on CSA and academic stress in the EFL context. According to their findings, EFL learners who presented high levels of coping style are more powerful in self-assessment and stress management.

#### Foreign Language Anxiety (FLA)

Academic anxiety is a general term that entails different anxieties that learners may experience in their educational lives (Cassady, 2010a). Based on this stipulation, learners’ anxiety (e.g., test anxiety, math anxiety, foreign language anxiety, and science anxiety) may hinder their academic progress (Cassady, 2010b). FLA was the target of the current study. FLA is a situation-specific phenomenon that triggers a low self-appraisal of communicative competencies in the language learning environment (Rodríguez & Abreu, 2003). Based on Horwitz et al. (1986), FLA entails three aspects: communication apprehension, test anxiety, and fear of negative evaluation. Communication apprehension refers to the anxiety that learners may experience while interacting with others or their problems in listening comprehension. The second aspect of FLA, test anxiety may happen if learners fear from failure in an examination. Fear of negative evaluation refers to uneasiness about other people’s judgments and avoiding situations that may trigger negative judgments of other people.

Various stimuli may evoke the students’ foreign language anxiety, such as students’ evaluation of their language aptitude, their personality traits, the language classroom experiences, their progress in language learning, and their reciprocal relationships with teachers and peers (Alamer & Almulhim, 2021). In the same line of inquiry, Brown et al. (2001) highlighted that learners’ personality types (introversion vs. extroversion) are major factors in shaping learners’ anxiety of foreign language classes. Based on Santos et al. (2021) and Burić et al. (2016), anxiety influences learners’ classroom interactions, learning and assessment, and in consequence formulate learners’ development and final growth. The Attentional Control Theory (ACT) explains anxiety and its negative effects (Eysenck et al., 2007). ACT is rooted in the processing efficiency theory (PET) proposed by Eysenck and Calvo (1992) and discusses that anxiety by absorbing threat-related stimuli harms attentional control. ACT also explains that anxious learners experience high levels of worry and low levels of self-confidence that leads to unsuccessful performance (Eysenck et al., 2007).

The literature on FLA portrays its interplay with other student-associated constructs. As an example, the findings of Zheng and Cheng (2018) witnessed the negative impacts of the learners’ anxiety on their language performance. Furthermore, Omidvar et al. (2013) confirmed that FLA decreased learners’ academic motivation in the EFL context. In the same vein, Camacho et al. (2021) conducted a study to inspect the effects of learners’ social support and anxiety on their academic motivation during COVID-19. As their outcomes indicated, the more students felt anxious, the less they were successful in their social relationships. In such a situation, they also felt less motivated to do their learning activities. Moreover, Bielak (2022) documented that FLA and foreign language enjoyment are the two critical factors determining L2 fluency. From another viewpoint, skill-based anxiety and its role in language learning were explored in recent studies. As an example, the listening anxiety (Zhang, 2013), speaking anxiety (Çağatay, 2015; Prentiss, 2021), reading anxiety (Hamada & Takaki, 2021a, 2021b), and writing anxiety (Zhang, 2019) were the target of these studies. According to the outcomes of the abovementioned studies, listening, speaking, reading, and writing anxiety were the major causes of students’ demotivation and failure. In a recent study, Fathi et al. (2021) asserted that FLA and grit could predict learners’ willingness to communicate (WTC) in an EFL context. Khajavy et al. (2021) also found that FLA, WTC, and enjoyment had reciprocal relationships. It means EFL learners’ enjoyment can foster their WTC, while FLA is considered an obstacle in this regard.

#### Objectives of the present study

Effective instruction and assessment are the major goals of successful education. Instruction and assessment work best when the both psychological and mental health of the learners are taken into consideration by policymakers, curriculum designers, and teachers. Learners also need to be armed with self-aid constructs to help them monitor themselves and make effective decisions, especially in the face of chaos and complexities. Via the lens of L2 grit and CSA, language learners are hoped to be able to overcome their FLA more successfully. As it was discussed before, the reciprocal relationship of the constructs was completely under shadow. The possible relationship between L2 grit and CSA or CSA and FLA was also untouched, particularly in the EFL context. Considering the attributions of these constructs in the realm of language learning as well as the paucity of research, this study sought to propose a model to portray the contribution of learners’ L2 grit and CSA to FLA. The outcomes of this study can be helpful theoretically and practically. It can increase the knowledge and awareness related to language instruction and assessment, and consequently, effective language learning is guaranteed. Having these standpoints in mind, the following research questions were raised:

- RQ1: How does EFL university learners’ L2 grit influence their CSA?
- RQ2: How does EFL university learners’ L2 grit influence their FLA?

According to these research questions, the following null hypotheses were formulated:

- H01. EFL university learners’ L2 grit does not influence their CSA.
- H02. EFL university learners’ L2 grit does not influence their FLA.

## Method

In this section, all the methodological steps that were involved in the current investigation are illustrated.

### Participants

The participants of this study were 418 university students, who were studying English Teaching (193), English Literature (93), and English Translation (132) at the BA level, at different universities in Iran. The criteria for choosing the participants were convenience or opportunity sampling procedures. The age range of the participants was between 18 to 23, and there were 175 males and 243 females.

### Instruments

#### The language-domain-specific grit scale (LDSGS)

The LDSGS designed and validated by Teimouri et al. (2020) was employed to gauge EFL university students’ grit. This scale includes 12 items: six items to assess perseverance of effort (e.g., I will not give up learning English until I master it) and six items to assess the consistency of interest (e.g., My interests in learning English changes from year to year) on a five-point Likert scale ranging from 1 “not at all like me” to 5 “very much like me.” The reliability of the (L2-Grit) estimated via Cronbach’s alpha (ranging from 0.825 to 0.911) was significant in this research.

#### The core of self-assessments questionnaire (CSAQ)

The university students’ fundamental self-assessments were assessed through CSAQ, developed and validated by Judge et al. (2003). This scale comprises 12 items in a 5-point Likert scale: strongly disagree (1), disagree (2), neutral (3), agree (4), and strongly agree (5). On this scale, the students’ scores ranged from 12 to 60. High scores on this scale reflected positive self-evaluation, while low scores reflected negative self-evaluation. In the present study, the reliability of CSEQ was 0.912, which indicated acceptable reliability.

#### The Foreign Language Classroom Anxiety Scale (FLCAS)

FLCAS, proposed and validated by Horwitz et al. (1986), was applied to inspect the level of anxiety that university students experience in their foreign language classroom. This scale involves 33 items in a five-point Likert scale (ranging from strongly agree to strongly disagree) to measure communication anxiety, fear of negative evaluation, test anxiety, and anxiety of foreign language class. Based on the report of Cronbach’s alpha, the reliability of the L2-Grit was acceptable (ranging from 0.811 to 0.892).

#### Procedures

This study was conducted via a web-based platform, which was started in March and ended in June 2022. The university students were asked to complete an electronic survey form, including the LDSGS, CSAQ, and FLCAS, through Google Forms. Based on the design of the electronic survey, each part in the electronic survey form should be necessarily linked; thus, no data were missed. The return rate was 85.9% and 418 forms were received. With the help of the electronic survey, researchers were able to collect data from different universities with varying age groups and fields of study.

#### Data analysis

Kolmogorov-Smirnov Test was employed to check the normal distribution of the data. Based on the result of the Kolmogorov-Smirnov test, the data were normally distributed. Therefore, confirmatory factor analysis (CFA), and SEM using linear structural relations (LISREL) 8.80 were employed to analyze the data. As Hair et al. (1998) highlighted, all the latent variables should be validated using CFA. Then, SEM was used. SEM is a robust multivariate procedure and it is used to take a confirmatory hypothesis-testing approach for the proposed structural theory (Schreiber et al., 2006). In SEM, the measurement model and the structural model were examined (Kunnan, 1998). The measurement model is used to investigate the association between the observed variables and latent variables. The structural model is applied to inspect the association between the latent variables.

## Results

In this section, the results of the statistical analysis are reported. In the following table, the descriptive statistics of university students’ L2-Grit, CSA, and FLA are presented.

As Table 1 displays, between the components of the L2-Grit, measuring perseverance of effort (*M*=22.313, SD=5.378) got the highest mean scores. Moreover, the mean score of CSA was (*M*=39.854, SD=9.590). Among the components of FLA, test anxiety presented the highest mean score (*M*=31.402, SD=5.606) and fear of negative evaluation got the lowest mean score (*M*=27.921, SD=7.118). Then, the normality distributions of the data were examined via the Kolmogorov-Smirnov Test. Moreover, Table 2 presents the results of the Kolmogorov-Smirnov Test.

**Table 1:** Descriptive statistics

		*N*	Minimum	Maximum	Mean	Std. Deviation
L2-Grit	Measuring perseverance of effort	418	6	30	22.313	5.378
	Consistency of interest	418	6	30	20.431	5.458
CSA	CSA	418	13	60	39.854	9.590
FLA	Communication anxiety	418	15	40	28.990	6.445
	Fear of negative evaluation	418	8	40	27.921	7.118
	Test anxiety	418	18	40	31.402	5.606
	The anxiety of foreign class	418	14	45	29.708	7.276

**Table 2:** The results of Kolmogorov-Smirnov test

Instrument	Subscales	Kolmogorov-Smirnov *Z*	Asymp. Sig. (2-tailed)
L2-Grit	Measuring perseverance of effort	0.884	0.415
	Consistency of interest	0.979	0.293
CSE	The Core of Self-evaluations Questionnaire (CSEQ)	0.803	0.539
FLA	Communication anxiety	1.026	0.243
	Fear of negative evaluation	0.824	0.505
	Test anxiety	0.911	0.378
	The anxiety of foreign class	0.925	0.359

According to Table 2, the sig value for all the instruments and their subscales were higher than 0.05, which indicated that the data were normally distributed. Thus, parametric methods were suggested to investigate the related research hypotheses. The LISREL 8.80 statistical package was used to inspect the structural relations between L2-Grit, CSA, and FLA. To evaluate the model fit, the chi-square magnitude, the root-mean-squared error of approximation (RMSEA), the Comparative Fit Index (CFI), and the Normed Fit Index (NFI) were utilized. The chi-square should be non-significant and the chi-square/df ratio should be lower than 3 (Jöreskog, 1990). The RMSEA is considered to be to be lower than 0.1 (Jöreskog, 1990). The NFI with a cut value greater than 0.90, The Good Fit Index (GFI) with a cut value greater than 0.90, and CFI with a cut value greater than 0.90 reflect a good fit (Jöreskog, 1990).

According to Table 3, the chi-square/df ratio (2.801) and the RMSEA (0.071) were also acceptable. The other three fit indices, GFI (0.923), NFI (0.951), and CFI (0.914), reached the acceptable fit thresholds.

**Table 3:** Fit indices (model 1)

Model				*RMSEA*	*GFI*	*NFI*	*CFI*
Cut value			>3	>0.1	>0.9	>0.9	>0.9
Model 1	374.60	132	2.838	0.066	0.942	0.953	0.912

**Fig. 2:**
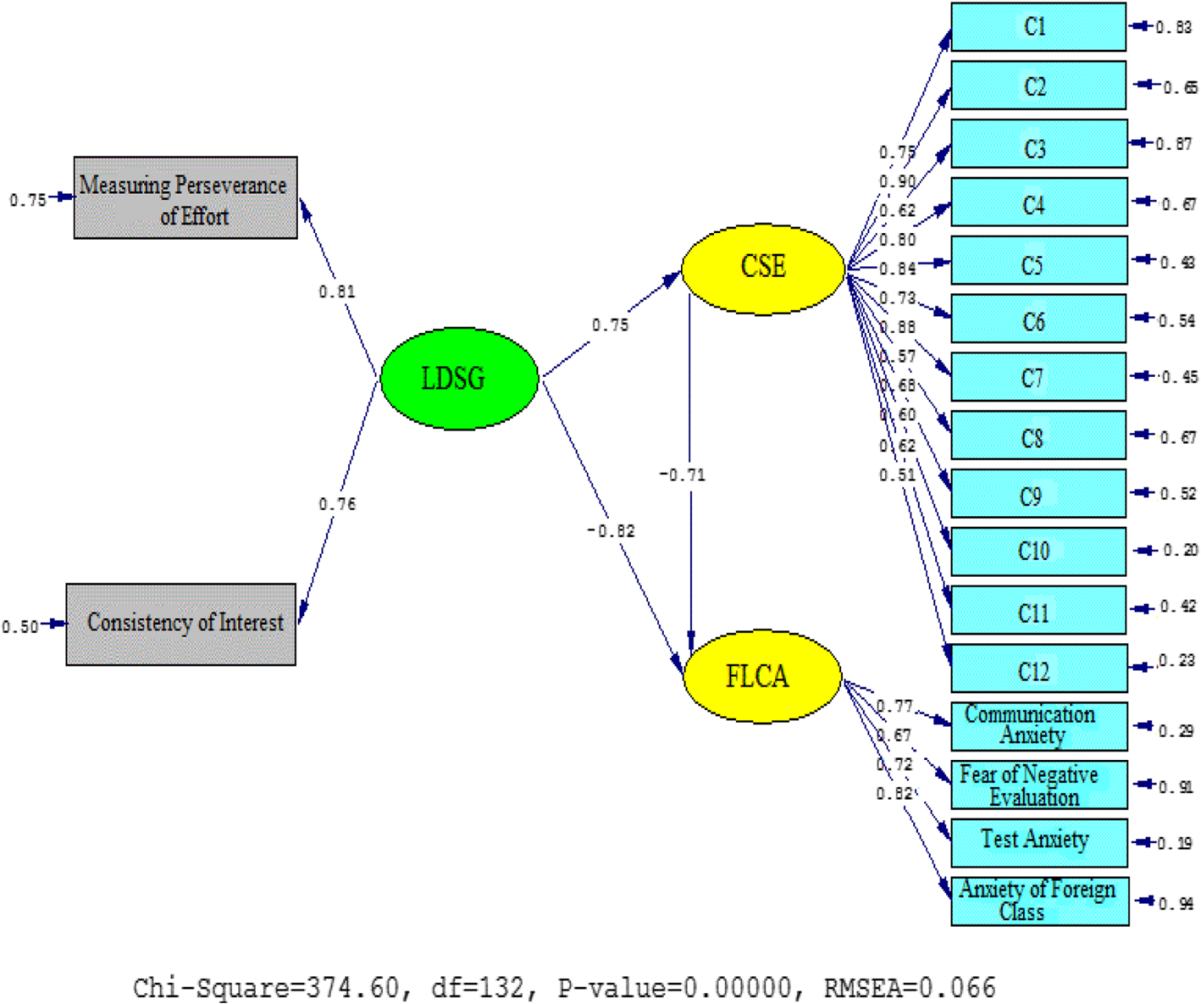
Schematic representation of path coefficient values for the relationships between L2-grit, CSA, and FLA (model 1)

**Fig. 3:**
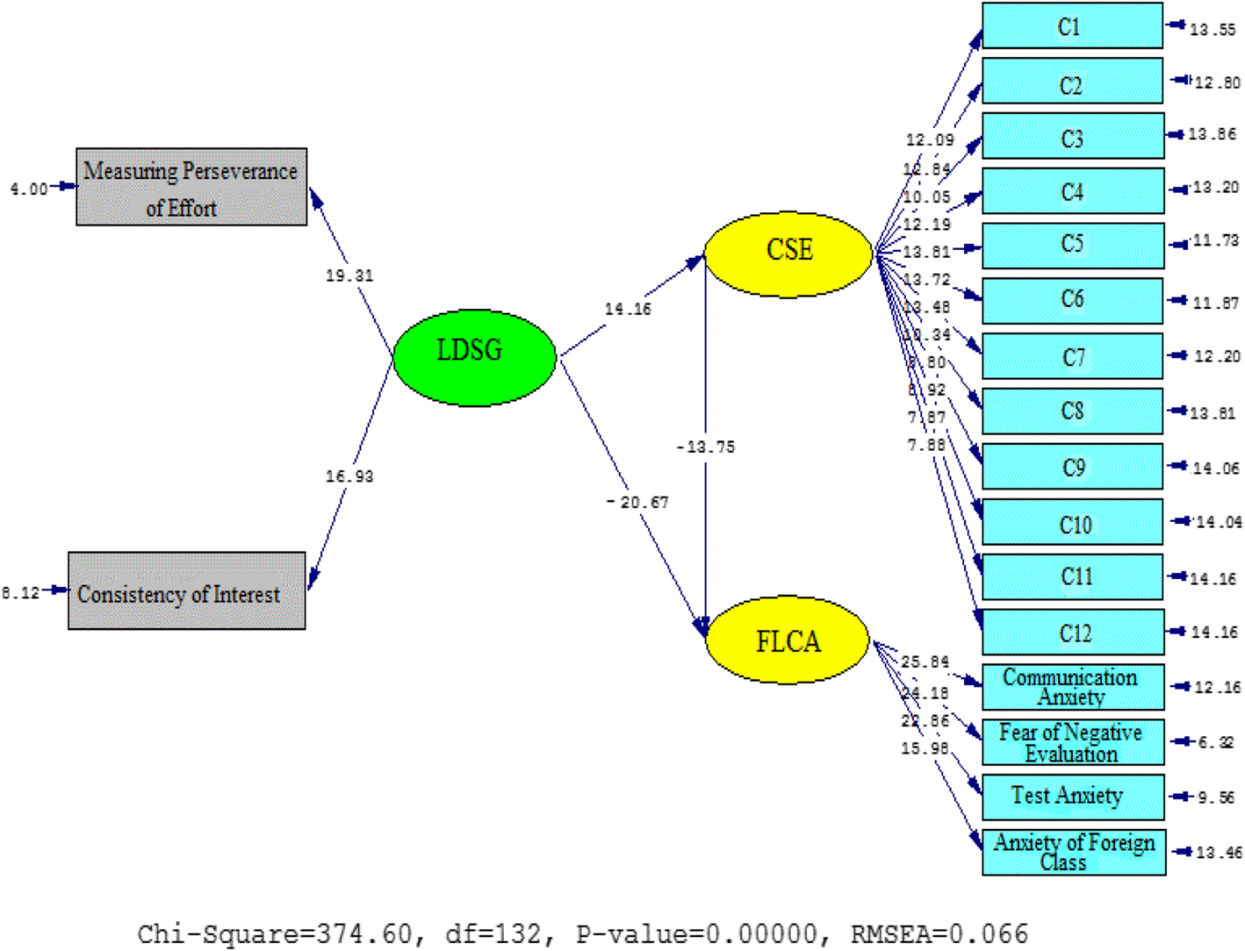
T Values for path coefficient significance (model 1)

The *t* values and standardized estimates were explored to check the strengths of the causal relationships among the variables. As Figs. 2 and 3 demonstrate, L2-Grit influenced students’ CSA (*β* = 0.75, *t* = 14.16) and FLA (*β* = −0.82, *t* = −20.67) significantly; the *t* value was greater than 1.96. The effect of CSA on FLA was significant and in a negative direction (*β* = −0.71, *t* =−13.75), and the *t* value was lower than −1.96.

According to Table 4, the fit indices in the second model were acceptable. It means the chi-square/df ratio (2.828) and the RMSEA (0.066) were acceptable. Additionally, GFI (0.924), NFI (0.932), and CFI (0.911) reached the acceptable fit thresholds.

**Table 4:** Fit indices (model 2)

Model				RMSEA	GFI	NFI	CFI
Cut value			>3	>0.1	>0.9	>0.9	>0.9
Model 2	2898.47	1025	2.828	0.066	0.924	0.932	0.911

**Fig. 4:**
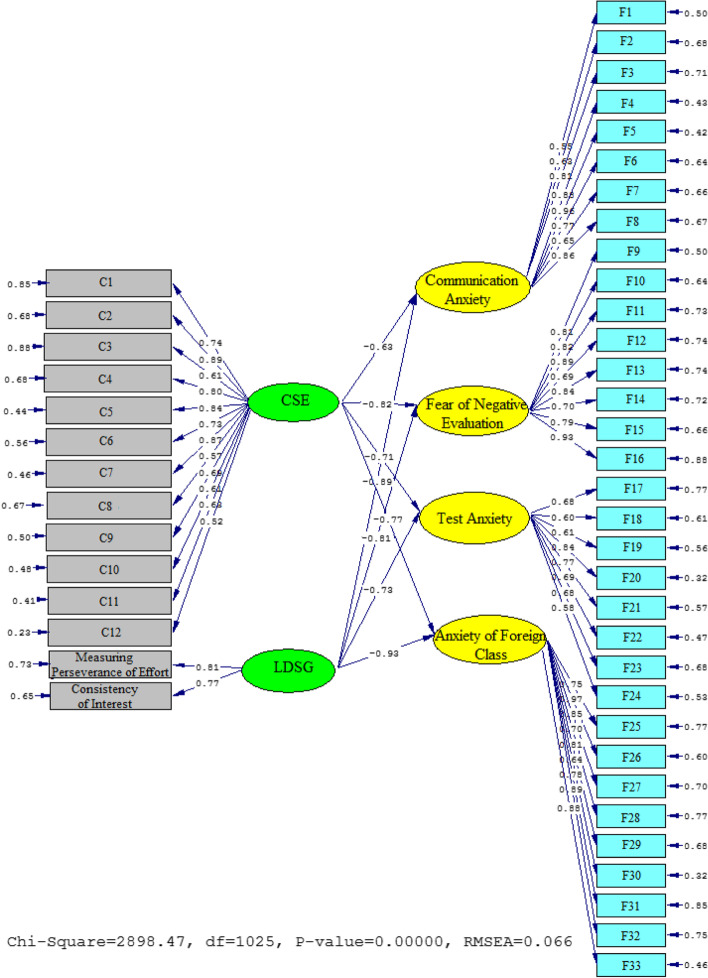
Schematic representation of path coefficient values for the influential role of L2-grit, CSA, and FLA (model 2)

**Fig. 5:**
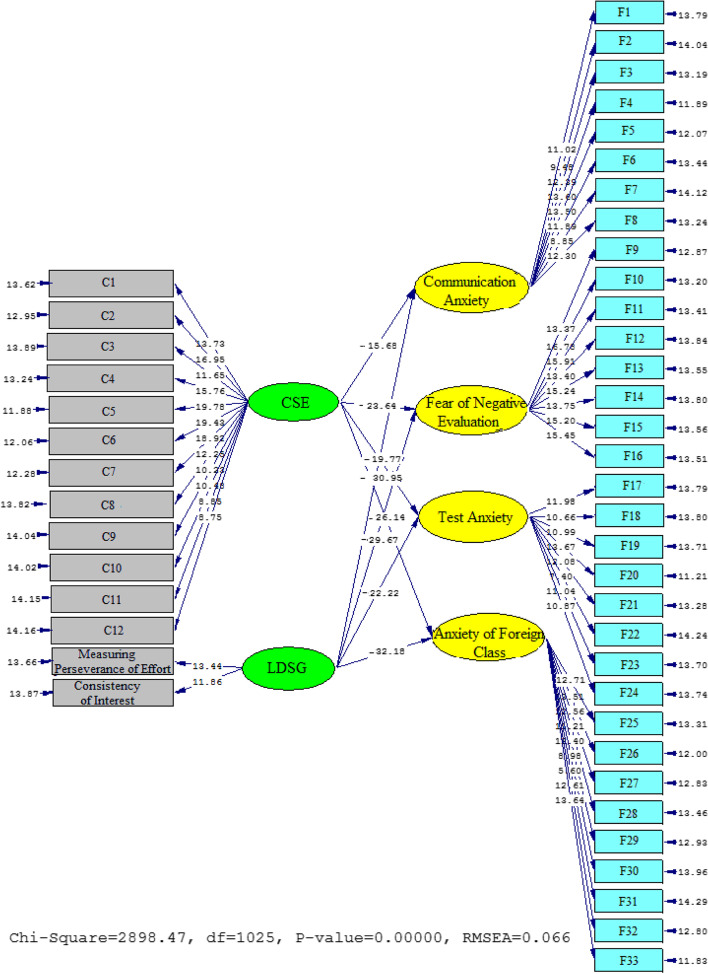
*T* values for path coefficient significance (model 2)

Figures 4 and 5 depict the schematic representation of path coefficient values for the relationship between L2-Grit, CSA, and FLA’s subscales. As the findings indicated, L2-Grit significantly and in a negative direction influenced communication anxiety (*β* = −0.89, *t* = −30.95), fear of negative evaluation (*β* = −0.81, *t* = −29.67), test anxiety (*β* = −0.73, *t* = −22.22), and FLA (*β* = −0.93, *t* = −32.18). Similarly, the relationship between CSA and the FLA’s subscales was significantly negative. That is, the effect of CSA on communication anxiety (*β* = −0.63, *t* = −15.68), fear of negative evaluation (*β* = −0.82, *t* = −23.64), test anxiety (*β* = −0.71, *t* = −19.77), and anxiety of foreign class (*β* = −0.77, *t* = −26.14) were significant and in a negative direction influence.

Furthermore, the association between L2-Grit, CSA, and FLA’s subscales was investigated via a Pearson product-moment correlation.

Based on Table 5, there were significant relationships between L2-grit and the subcomponents of FLA as follows: communication anxiety (*r*= −0.912, *p*<0.01), fear of negative evaluation (*r*= −0.832, *p*<0.01), test anxiety (*r*= −0.754, *p*<0.01), and anxiety of foreign class (*r*= −0.952, *p*<0.01). Regarding the relationship between CSA and the subcomponents of FLCAS, the outcome was as follows: communication anxiety (*r*=−0.635, *p*<0.01), fear of negative evaluation (*r*=−0.841, *p*<0.01), test anxiety (*r*=−0.748, *p*<0.01), and anxiety of foreign class (*β* = −0.798, *t* = *p*<0.01).

**Table 5:** The correlation coefficients among L2-Grit, CSA, and FLA’s subscales

	L2-Grit	CSA	Communication anxiety	Fear of negative evaluation	Test anxiety	Anxiety of foreign class
L2-Grit	1					
CSA	0.795^a^	1				
Communication anxiety	−0.912^a^	−0.635^a^	1			
Fear of negative evaluation	−0.832^a^	−0.841^a^	0.776^a^	1		
Test anxiety	−0.754^a^	−0.748^a^	0.731^a^	0.643^a^	1	
The anxiety of foreign class	−0.952^a^	−0.798^a^	0.582^a^	0.556^a^	0.631^a^	1

^a^Correlation is significant at the 0.01 level (2-tailed)

## Discussion

This study intended to uncover the interplay between learners’ L2 grit, CSA, and FLA in the EFL context. This goal was reached by applying a structural equation modeling approach to build a causal structural model, which can portray the relationships between the learners’ L2 grit, CSA, and FLA. As model 1 and model 2 depicted, L2 grit and CSA predict FLA. Furthermore, the mediator role of L2 grit on CSA was pictured in model 1. Therefore, the first null hypothesis (H01. EFL university learners’ L2 grit does not influence their CSA.) and second null hypothesis (H02. EFL university learners’ L2 grit does not influence their FLA.) was rejected, and it can be implied that learners’ L2 grit, CSA, and FLA are interrelated.

Considering the first research question (RQ1: How does EFL university learners’ L2 grit influence their CSA?), the findings of this research indicated that EFL learners’ L2 grit could predict their CSA (Model 1). That is, the synthesis of perseverance of effort and consistency of interest enhance EFL learners’ CSA. It can be inferred that grittier EFL learners are consistent in their attempts to reach their goals. The more they are consistent in their efforts and goals, the better they can evaluate themselves. As it was mentioned before, the CSA is a combination of self-esteem, generalized self-efficacy, neuroticism, and locus of control. It can be concluded that the grittier learners reach higher levels of CSA; thereby, they develop higher levels of self-esteem, self-efficacy, neuroticism, and locus of control (Model 2).

As the existing literature on L2 grit and CSA echoes, to date, no identical studies have been conducted to gauge the possible interplay between them. Previous studies highlighted that language learning is a cognitive activity that asks for active and engaged participants (Yang, 2021; Zabihi, 2018; Zhao & Liao, 2021). Shafiee Rad and Jafarpour (2022) evidenced that learner L2 grit, emotion regulation, and resilience are correlated. Their findings reflected that L2 grit helps EFL learners evaluate and modify their emotions and also recover quickly from difficulties. In addition, Abdolrezapour and Ghanbari (2021) as well as Khajavy and Aghaee (2022) found out EFL learners’ L2 grit and emotion predict the academic achievement of the learners. In the same line of inquiry, the relationships between L2 grit and self-efficacy as one aspect of CSA were confirmed (Yang et al., 2022). The findings of Jahara et al. (2022) supported this finding. As they concluded, learners’ coping styles predict their abilities in CSA.

More precisely, this result can be supported by the underpinning theories of learner L2 grit. Duckworth (2016) characterized L2 grit as learners’ enthusiasm and long-lasting attempts. Additionally, in the model of the language-domain-specific grit by Teimouri et al. (2020), the emphasis is on language learners’ perseverance of effort and consistency of interest. Thus, it can be implied that when EFL learners have positive attitudes toward education and clear goal for their progress, they attempt to critically assess their activities in order to find and modify their learning progress. The CSA, from another perspective, helps learners to evaluate the assessment procedures, which is an inevitable part of learning procedures.

The other outcome of the present study was related to the second research question (RQ2: How does EFL university learners’ L2 grit influence their FLA?). The study finding mirrored that EFL learners’ L2 grit had predictive power on FLA (Model 1). This relationship was significantly negative. It shows that the more learners improve the level of their grit, the better they can manage the experienced anxiety in language classes. Theoretically, this finding can be discussed that the high levels of perseverance of effort and consistency of interest (Teimouri et al., 2020) among university students act in favor of their attentional control as well as self-confidence in provoking experiences that may lead to anxiety (Eysenck et al., 2007). Furthermore, the findings indicated that EFL learners’ grit tendencies significantly and negatively correlated with the subcomponents of FLA. It means L2 grit empowers learners to supervise their communication anxiety, fear of negative evaluation, test anxiety, and anxiety of foreign language class (Model 2). This result is in accord with the previous studies though limited, which highlighted the key role of L2 grit on the regulation of emotions in general and anxiety in particular (Khajavy, 2021; Khajavy & Aghaee, 2022; Wei et al., 2019). The finding of Fathi et al. (2021) in the EFL context was also another support for the critical role of L2 grit in decreasing learners’ anxiety and increasing their willingness to communicate.

It was also discovered that CSA has a mediator role in the learners’ foreign language anxiety (model 1). Based on the finding of this study, the more the learners critically evaluate themselves, the less they suffer from anxiety in language classes. It is inferred that through the lens of self-assessment, learners fundamentally assess and evaluate themselves. This evaluation may not be possible without learners’ higher-order thinking skills, self-efficacy beliefs, and positive self-esteem. According to Af Ursin et al. (2021) and Russell (2020), if learners have high expectations beyond their abilities, they will experience anxiety. Therefore, EFL learners need to be equipped with self-assessment to effectively regulate their unpleasant emotions, such as anxiety in the educational context (Aghili Mehrizi et al., 2022; Kuchkarova, 2022).

## Conclusion

This study pined the effective role of L2 grit on EFL learners’ CSA and FLA. The findings of this study add a strong empirical confirmation that L2 grit is crucial in directing learners’ CSA and managing language learning anxiety. L2 grit can be assumed as a key on the road to learners’ educational progress, which helps them to be involved and consistent for a longer period of time, even in language learning chaos and complexities. Additionally, this study pictures the mediator role of the CSA in language learner anxiety. Their relationships were directed negatively. That is, the higher the levels of self-assessment, the lesser the possibility of anxiety experiences. It can also be concluded that L2 grit and CSA open the minds of the learners, especially university students to work on the problems and overcome FLA. All in all, the study is among the first steps to uncovering the reciprocal relationships between these variables. As it seems, this domain is still in its infancy and asks for more empirical studies to brighten the road, which promotes the learners’ academic achievement and guarantees effective pedagogy.

### Implications of the study

This study proposes some pedagogical implications for language instructors, learners, and curriculum designers. To increase the efficiency of learning and assessment, instructors, learners, and curriculum designers need to acquire related knowledge about personality and situational determinants of L2 grit and CSA. Fear of speaking, listening, reading, and writing a foreign language is common among learners. Instead of dwelling on the errors made and negative thoughts, learners should learn how to manage their fear and improve their skills to perform better in the next assessment tasks. Training courses and designing plans are needed to focus on learning and practicing the implementations of L2 grit and CSA as vital aspects of learners’ traits, in particular university learners. To this end, pre-service and in-service training programs are suggested to design special programs for language teachers and university professors to learn how they can improve L2 grit and self-assisted constructs among their learners. As different ups and downs in language learning may trigger learners’ anxiety, teachers are expected to help their learners to modify and regulate their unpleasant emotional experiences. In so doing, providing some training programs for teachers as well as learners would be of great help. It is also necessary to include some major sections in the design of the curriculum and language learning syllabus to deal with self-assisted constructs with the prospects of effective instruction and assessment. These helpful strategies can also be considered in materials development and designing assessment tasks.

### Limitations and suggestions for future researchers

Similar to other studies, the findings of this study should be considered with some limitations: Firstly, this research was quantitative in nature. Future research may employ qualitative or mixed method approaches to inspect the interplay between L2 grit, the core of self-assessment, and foreign language anxiety. Secondly, the association of these variables with other teacher-related constructs (e.g., autonomy, self-regulation, academic buoyancy, and emotion regulation) can be regarded for future studies. Thirdly, the possible influence of demographic variables of the participants on their L2 grit, the core of self-assessment, and foreign language anxiety was not the target of the current research. Future research can concentrate on these variables to see how they affect learners’ their L2 grit, CSA, and FLA. The other limitation of this study was related to how the participants were chosen. Because of practical constraints, convenience sampling or opportunity sampling procedures were applied in our research, which may not be representative. In the future, researchers can use other methods of data gathering to ensure the generalizability of the findings. Furthermore, the possible effect of the learners’ sociocultural background was not the target of this study. Future research can concentrate on this aspect and do further research in this regard. In addition, the effects of teachers’ L2 grit, self-assessment, and anxiety on their learners’ L2 grit, self-assessment, and anxiety can be considered a future research avenue. Lastly, our study was conducted among EFL university students; it is suggested to investigate the reciprocal associations between these variables in other educational contexts, such as schools and language institutes.

## Data Availability

The authors state that the data supporting the findings of this study are available within the article.

## Abbreviations

CFA: Confirmatory factor analysis
CFI: Comparative Fit Index
CSA: Core of self-assessment
CSAQ: Core of self-assessments questionnaire
EFL: English as a Foreign Language
FLA: Foreign language anxiety
FLCAS: Foreign Language Classroom Anxiety Scale
GFI: Good Fit Index
LDSG: Language-domain-specific grit scale
LISREL: Linear structural relations
NFI: Normed Fit Index
RMSEA: Root-mean-squared error of approximation
SEM: Structural equation modeling
